# Influence of microenvironment on engraftment of transplanted β-cells

**DOI:** 10.3109/03009734.2010.548609

**Published:** 2011-02-11

**Authors:** Per-Ola Carlsson

**Affiliations:** Department of Medical Cell Biology, Uppsala University, Uppsala, Sweden, and Department of Medical Sciences, Uppsala University, UppsalaSweden

**Keywords:** Engraftment, implantation site, islet transplantation

## Abstract

Pancreatic islet transplantation into the liver provides a possibility to treat selected patients with brittle type 1 diabetes mellitus. However, massive early β-cell death increases the number of islets needed to restore glucose homeostasis. Moreover, late dysfunction and death contribute to the poor long-term results of islet transplantation on insulin independence. Studies in recent years have identified early and late challenges for transplanted pancreatic islets, including an instant blood-mediated inflammatory reaction when exposing human islets to the blood microenvironment in the portal vein and the low oxygenated milieu of islets transplanted into the liver. Poor revascularization of remaining intact islets combined with severe changes in the gene expression of islets transplanted into the liver contributes to late dysfunction. Strategies to overcome these hurdles have been developed, and some of these interventions are now even tested in clinical trials providing a hope to improve results in clinical islet transplantation. In parallel, experimental and clinical studies have, based on the identified problems with the liver site, evaluated the possibility of change of implantation organ in order to improve the results. Site-specific differences clearly exist in the engraftment of transplanted islets, and a more thorough characterization of alternative locations is needed. New strategies with modifications of islet microenvironment with cells and growth factors adhered to the islet surface or in a surrounding matrix could be designed to intervene with site-specific hurdles and provide possibilities to improve future results of islet transplantation.

**Figure:**
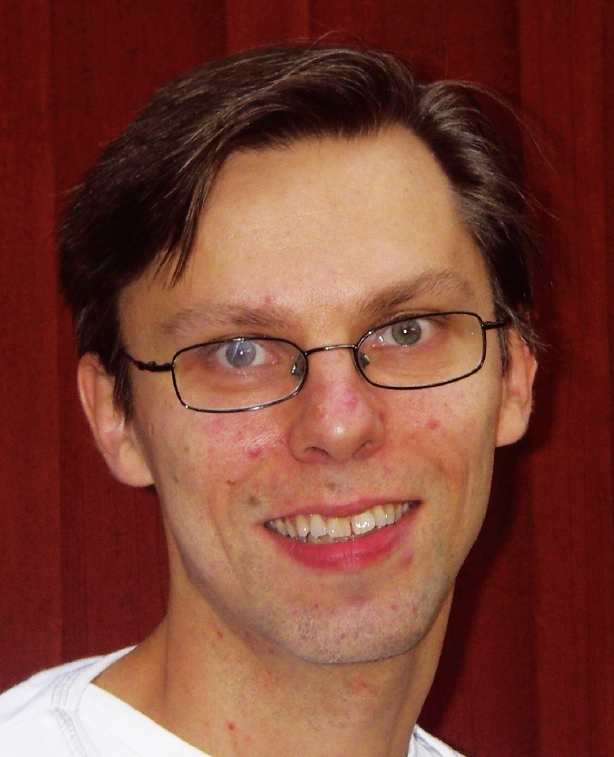
Winner of the Eric K. Fernström Award for young investigators, 2010 at the Medical Faculty of Uppsala University.

Clinical pancreatic islet transplantation has, since the introduction of the concept, been almost exclusively performed through the intraportal route to the liver based on the pioneering work of the late Dr Paul Lacy (1). Early results of allogeneic islet transplantation were poor, with insulin independence 1 year post-transplantation less than 10% (2). However, in more recent years, transplantation of pancreatic islets from deceased donors has become a curative treatment for selected patients with type 1 diabetes (3,4). Nevertheless, many problems still remain, including massive early islet cell death (5). The intravascular injection of pancreatic islets has been shown to elicit an instant blood-mediated inflammatory reaction (IBMIR) triggered by tissue factor and cyto/chemokines expressed by pancreatic islets in the whole blood microenvironment of the portal vein (6,7). Moreover, the liver tissue has a very low oxygen tension (8,9), high numbers of resident macrophages (Kupffer cells) (10), and possibly also high concentrations of immunosuppressive drugs exposing the islet cells following intestinal uptake of the drugs. This means that in most cases at least two donor pancreases are needed to restore glucose homeostasis, which is far more than the alleged 10%–20% of the total islet volume suggested to be enough to maintain normoglycaemia in humans (3). Despite the large transplanted islet mass, the functional capacity of the transplanted islets has been shown to correspond to only about 20% of that found in a non-diabetic person (11). Within the first years post-transplantation most patients revert to insulindependence if not re-transplanted. However, only low doses of exogenous insulin are then usually needed, and the major problems with hypoglycaemic attacks most often no longer exist. It is presently unknown whether the progressive decline in islet graft function is site-specific or applies also to other implantation sites.

## Challenges for pancreatic islets early after transplantation

**Figure 1.**
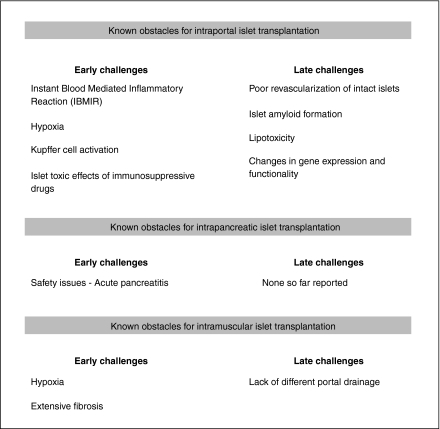
Obstacles for successful pancreatic islet transplantation at different sites.

A thrombotic reaction and complement cascade is elicited when human islets are exposed to blood, which is manifested by islet entrapment in blood clots, leucocyte infiltration, predominantly neutrophil granulocytes, and disruption of the islet morphology (12) (Figure 1). This so-called IBMIR occurs in clinical islet transplantation as shown by an increase in concentrations of thrombin–antithrombin complex immediately after islet infusion, and the parallel increase in c-peptide release indicates massive β-cell death (7). A study by combined positron emission tomography and computed tomography (PET/CT) technique indicates that early islet cell death can be estimated to be approximately 25% in patients intraportally transplanted with islets (13). Besides innate immune reactions, islets transplanted to the liver may have restricted survival rates due to the low oxygen tension in the liver parenchyma. Experimental studies indicate that approximately 70% of the islets are hypoxic 1 day after intraportal transplantation, as evaluated by the biochemical marker pimonidazole which accumulates in islet cells at oxygen tension levels below 7.5–10 mmHg (14). Since this marker is not accumulated in dead or dying cells, the number of hypoxic cells may even be under-estimated. Clinically, also high concentrations of immunosuppressive drugs in the portal vein are likely to hamper islet survival by direct toxic effects (15,16).

Experimentally, several sites besides the liver have been tested for islet transplantation, including the subcutis, muscle, the intraperitoneal site, the renal subcapsular site, the spleen, bone-marrow, pancreas, and the omental pouch. A more thorough characterization of engraftment has recently been performed with regard to implantation of islets into the pancreas or muscle. Orthotopic implantation of islets, i.e. into the pancreas, has been described in experimental settings (17,18). Although being the most physiological environment for islets, the pancreas has rarely been considered as a potential implantation organ in clinical practice. Surgical interventions and injections in the pancreas are difficult, and there is a high risk of acute complications due to leakage of enzymes from the exocrine cells that causes tissue damage and inflammation. Clearly, implantation techniques need to be improved to minimize these early risks, e.g. by subcapsular implantation of islets or other techniques. Safety issues have also to be carefully investigated in large-animal models. Nevertheless, studies in small animals indicate that this site provides good conditions for early survival of implanted islets, with minimal inflammatory and fibrotic components (19). Moreover, the oxygenation of the pancreas is much better than the liver, with a mean oxygen tension in the exocrine parenchyma of ∼30 mmHg (20).

Striated muscle has clinically been tested as implantation site for pancreatic islets based on the feasibility of this site for autotransplantation of parathyroid glands and its easy access. In one Swedish patient, islets were, due to severe hereditary chronic pancreatitis, autotransplanted intramuscularly with a remaining high and stable c-peptide production (21). However, also this site seems to provide challenges for early survival of the islets based on the extensive fibrosis occurring in the islet grafts (21,22). Especially prominent central necrosis parts may occur in islet grafts, if islets are implanted as clusters, and cause central fibrosis (23).

## Challenges for pancreatic islets late after transplantation

For pancreatic islets to survive and regain function after transplantation they need to be properly revascularized. Experimental studies show that mouse and human islets transplanted intraportally are poorly revascularized, similar to islets implanted subcapsularly to the kidney or spleen (24–26). It is noteworthy that endothelial cells seem to be stimulated to grow towards the implanted islets, i.e. form a dense vasculature in the immediate vicinity of the islets, but few blood vessels enter the endocrine parenchyma. This is reflected in an impaired oxygenation of intraportally implanted islets that remains for at least 1month post-transplantation, and that is accompanied by increased apoptosis rates (Olsson R, Olerud J, Pettersson U, and Carlsson P-O; unpublished observation). Late maturation of the blood vessel system may occur, since numbers of pimonidazole-positive islets, suggesting prevailing hypoxia, decrease with time, and apoptosis rates become similar to those in endogenous islets at 3 months post-transplantation. Consequences of low blood vessel numbers for islets are not restricted merely to impaired oxygen and nutrient transport but also include less influence of paracrine supporting signals from islet endothelial cells on β-cell function and growth. Islet endothelial cells may normally support nutrient-stimulated insulin release and β-cell differentiation by their secretion of basement membrane components, especially laminin, and the glycoprotein thrombospondin-1 sequestered to the islet matrix (27,28). Their secretion of laminin and hepatocyte growth factor is of importance to maintain β-cell proliferation and increase the β-cell mass when functionally demanded (29,30). Impaired drainage of islet hormones induced by a no-flow phenomenon in isolated islets and early after transplantation also predisposes for sequestration of islet amyloid polypeptide (IAPP) as amyloid in islets. Irrespective of mechanism, amyloid formation has been described to occur in isolated human islets during culture (31) and following experimental transplantation to the liver in mice (32). A recent autopsy report of an islet-transplanted patient who died from a myocardial infarct indicates that amyloid forms following clinical islet transplantation to the liver (33). Late failure of islets transplanted to the liver may also be caused by insulin-induced intense lipogenesis in nearby hepatocytes. Proof of principle of improved long-term graft function has been shown in a rodent model by blocking lipogenesis (34). Hepatic steatosis has been reported in the clinical situation after islet transplantation (35,36). There are reports on site-specific alterations in islet function after intraportal islet transplantation as well, such as defective glucagon response to hypoglycaemia (37,38) caused by increased glucose flux and glucose levels within the liver secondary to increased glycogenolysis caused by systemic hyperglycaemia (39), and substantial changes in the β-cell gene expression and function (40,41). A perturbed glucose-stimulated insulin release in intrahepatic islets has been shown to correlate with defects in glucose oxidation, (pro)insulin biosynthesis, and decreased insulin content and associated with decreases in the islet gene expression of the β-cell differentiation marker pancreatic and duodenal homeobox gene-1 (PDX-1) and key enzymes in glucose transport and metabolism in the β-cells, such as glucose transporter-2, glucokinase, pyruvate carboxylase, and mitochondrial glycerol phosphate dehydrogenase (41).

Pancreatic islets experimentally transplanted to the pancreas are much better revascularized than intrahepatic islets (19). However, despite being implanted into their normal physiological microenvironment, intrapancreatically transplanted islets display some functional and gene expression changes, although not as profound as those observed in islets implanted in the liver. A characteristic finding for intrapancreatically transplanted islets seems to be their higher insulin release compared with endogenous islets at low glucose concentrations, and their high expression of the lactate dehydrogenase-A gene, which is normally expressed in low levels in β-cells (41). This may explain why animals receiving large numbers of intrapancreatically transplanted islets are slightly hypoglycaemic (18).

Pancreatic islets transplanted to striated muscle are rapidly and much better revascularized than intraportally transplanted islets (23). This seems to be irrespective of whether the islets are implanted in clusters or as single islets (22) and has been confirmed in patients autotransplanted with islets due to exocrine pancreas disease (23). The functionality of the newly formed capillaries is good, and oxygen tension levels in intramuscular islet grafts were only slightly decreased when compared to native islets (22). Despite lack of direct portal drainage, pancreatic islets implanted to muscle seem to have a superior function when compared to intrahepatic islets (23), although a prerequisite for this is the avoidance of cluster formation at islet implantation (42).

## Tentative strategies for islet microenvironment modification to improve islet graft survival and function

### Modifications of islet microenvironment in the liver

Recent characterization of islet engraftment at the intraportal site has identified numerous problems that need to be targeted to optimize graft survival and function. However, since islets are dispersed and embolized deep into the liver tissue at transplantation, strategies to modify liver microenvironment per se are difficult. Instead such modifications must include the systemic blood environment, or modifications of islet tissue or surfaces prior to transplantation. In fact, several strategies in order to intervene with IBMIR have successfully been tested experimentally *in vitro* and/or *in vivo* and include modifications of blood with thrombin inhibition by megalatran (43), or with low-molecular-weight dextran sulfate (44), or modifications of the islets and their surfaces with nicotinamide (45), heparin (46), or endothelial coating (47). At present, a clinical trial is conducted in the Nordic countries investigating the effects of low-molecular-weight dextran sulfate on outcome in patients transplanted intraportally with islets.

Different anti-apoptotic strategies have been tested to improve early islet survival in experimental models. Most successful studies include gene transfection strategies difficult to implement in the clinical situation. However, besides this, e.g. different caspase inhibitor therapies of recipients (48), or even of islets for transplantation (49), have proved effective in improving outcome in minimal islet mass models in mice. Moreover, merely prolactin or glucocorticoid supplementation of the culture medium has been shown to improve β-cell survival during human islet culture and early after experimental islet transplantation (50–52). Also long-acting glucagon-like peptide 1 analogues improve human islet survival in culture (53), protect murine β-cells of intraportal islet transplants from apoptosis (54), and are beneficial for glucose homeostasis in marginal mass islet-transplanted mice (55). One of these long-acting glucagon-like peptide 1 analogues, exenatide, has been introduced in clinical islet transplantation protocols at some transplantation centres (56,57).

Our recent studies indicate that damage to islets at intraportal transplantation may facilitate subsequent islet revascularization, whereas islets with maintained integrity are poorly revascularized and blood-perfused (Henriksnäs J, Lau J, Zang G, Berggren P-O, Köhler M, and Carlsson P-O; unpublished observation). Different means to improve islet revascularization have been tested including over-expression of the pro-angiogenic factor vascular endothelial growth factor (VEGF) in the islet tissue by virus vectors (58). More recently, a non-viral gene delivery approach has been successfully tested in a small-animal model, where VEGF was delivered to intraportally transplanted human islets by ultrasound-targeted microbubble destruction (59). Translation into the clinical situation may, however, be hampered by technique limitations in penetration of ultrasound in larger organs. More feasible in the clinical setting than trying to increase the expression of pro-angiogenic factors in the islet tissue may be to inhibit angiostatic factors. We recently showed proof of principle by inhibition of the angiostatic factor thrombospondin-1 in islets by a siRNA technique, which improved both islet graft revascularization and function (60). Unfortunately, at present there are no pharmacological receptor antagonists for thrombospondin-1 available. However, prolactin was found to decrease the expression of thrombospondin-1 in islets in culture and had beneficial effects for islet graft revascularization and function both when injected to recipient animals during the first days post-transplantation, or merely added during culture of islets prior to transplantation (50).

Cell coating provides interesting aspects to bioengineering of islets and to obtaining multiple effects that can diminish adverse events following transplantation. Besides previously mentioned endothelial coating, other cell types might be considered for modification of islet surfaces. As reported in a paper in the present issue of this journal, mesenchymal stem cells (MSCs) form an interesting approach to provide local immunosuppressive effects, at least early after transplantation (61). Positive effects of MSCs on revascularization in experimentally transplanted islets have recently been shown *in vivo* (62), and these cells can facilitate endothelial coating of the islet surface *in vitro* as well (63). Several other cell types are of interest to include in the islet microenvironment, including endothelial progenitor cells and neural crest stem cells. Endothelial progenitor cells seem to be able to diminish innate immune reactions and improve survival, revascularization, β-cell proliferation, and function in islet grafts (64). Neural crest stem cells have the ability to increase substantially β-cell proliferation and improve islet graft function (65).

### Modifications of islet microenvironment in extrahepatic sites

As described above, extrahepatic sites to some part provide different challenges for survival and regain of function when compared to the liver. There are also site-specific differences to consider. In general, however, in most cases transplantation to these sites does not include infusion of islets into blood, which diminishes IBMIR. Some innate immune reactions may remain, but the extent of these remains to be elucidated. It can be envisaged that hypoxia in newly transplanted avascular cells provides a limitation also in extrahepatic sites, especially when islets are implanted in clusters. Dispersion of islets at transplantation is of great advantage, and techniques for optimal implantation of islets need to be developed. Modifications of islet microenvironment can at most of these sites be obtained by the use of scaffolds and biomaterial implanted before or together with the islets in order to provide spatial and functional support for the islets (66). The biomaterials can be biodegradable or not, and can also be modified e.g. by inclusion of oxygen carriers such as polymerized haemoglobin, anti-inflammatory components or cells, and growth factors and cells to stimulate angiogenesis. In this manner, also substances and cells that do not readily adhere to islet surfaces can be used to modify islet microenvironment and create new micro-organs. Studies are on-going to optimize such micromilieus.

## Conclusions

In recent years, several hurdles that restrict the success of pancreatic islet transplantation into the liver have been identified. Possible solutions to some of these problems have also been tested and include interventions to the IBMIR and means to improve revascularization. In parallel, there has been an increased interest in change of implantation site in order to avoid the site-specific problems of the intraportal route. Most alternative implantation sites have, however, not been well characterized with regard to engraftment of islets, and many unidentified obstacles at alternative locations may remain. New strategies with modifications of the islet microenvironment with cells and growth factors adhered to the islet surface or in a surrounding matrix provide possibilities to improve results of islet transplantation irrespective of implantation site. Such technologies may also pave the way for future improvements of the maturation and function of stem cells as a surrogate for native β-cells *in vivo*.
